# Prognostic factors in elderly patients with T1 glottic cancer treated with radiotherapy

**DOI:** 10.1038/s41598-021-96146-5

**Published:** 2021-09-06

**Authors:** Anna Mucha-Małecka, Krzysztof Małecki, Natalia Amrogowicz, Beata Biesaga, Maciej Modrzejewski

**Affiliations:** 1 Department of Radiotherapy, Maria Sklodowska-Curie National Research Institute of Oncology, Cracow Branch, Garncarska 11, 31-115, Cracow, Poland; 2 Department of Radiotherapy for Children and Adults, University Children’s Hospital of Cracow, Wielicka 265, 30-663 Cracow, Poland; 3 Faculty of Health Sciences, Jagiellonian University in Cracow, Michałowskiego 12, 31-126 Cracow, Poland; 4 1St Radiation and Clinical Oncology Department, Maria Sklodowska-Curie National Research Institute of Oncology, Gliwice Branch, Wybrzeże Armii Krajowej 15, 44-101, Gliwice, Poland; 5 Center for Translational Research and Molecular Biology of Cancer, Maria Sklodowska-Curie National Research Institute of Oncology, Gliwice Branch, Wybrzeże Armii Krajowej 15, 44-101, Gliwice, Poland; 6 Department of Tumour Pathology, Maria Sklodowska-Curie National Research Institute of Oncology, Cracow Branch, Garncarska 11, 31-115, Cracow, Poland; 7 Department of Otolaryngology, Head and Neck Surgery, 5th Military Hospital with Polyclinic, Wroclawska 1-3, 30-901, Cracow, Poland

**Keywords:** Cancer, Oncology

## Abstract

The aim of the study was the evaluation of the effectiveness of radiotherapy in elderly T1 glottic cancer patients and prognostic factors with particular focus on comorbidities. Five-year overall survival, disease-specific survival, and local control rates were 63%, 92%, and 93%, respectively. Multivariate analysis showed that the following factors had statistically significant impact on local relapse risk and cancer death risk: diabetes, underweight, and fraction dose of 2 Gy. High number of comorbidities, high CCI, and underweight negatively influenced overall survival. A retrospective analysis was performed in a group of 131 T1N0M0 glottic cancer patients aged 70 and above treated with irradiation at the National Institute of Oncology in Cracow between 1977 and 2007. In the analyzed group men prevailed (92%) of mean age of 74 years. Each patient was diagnosed with at least one comorbidity with the following comorbid conditions being most frequent: hypertension, ischemic heart disease, and chronic obstructive pulmonary disease. In the studied group, the effect of comorbidities on overall survival was evaluated using Charlson Comorbidity Index (CCI). Twenty five (19%) patients showed underweight. All patients were irradiated once daily, 5 days a week, to a total dose of 60–70 Gy with a fraction dose of 2 or 2.5 Gy. Radiotherapy is an effective treatment modality in elderly T1 glottic cancer patients. Diabetes as comorbidity, underweight, and conventional dose fractionation decrease the probability of curative effect of radiotherapy in this group of patients, while high number of comorbidities diminishes the probability of long-term survival.

## Introduction

The incidence of cancer is rising in conjunction with expanding human lifespan. The risk of laryngeal cancer increases with age, reaching highest rates in the 7th decade in men (45/10^5^) and in the late 5th/early 6th decade in women (6/10^5^). In Poland in 1980, 2086 new cases of laryngeal cancer were reported, whereas in 2015 this figure rose to 2526 cases, including 587 (23%) patients aged 70 and above^1^. In patients with early glottic cancer two equally efficient treatment modalities (radiotherapy and surgery) are applied, leading to 5-year local control rates between 80 and 100%^2–8^. Treatment outcomes of radiotherapy in elderly individuals with early glottic cancer are similar to younger age groups^8^. However, in the majority of patients comorbidities are present that may be contributing to lower performance status and overall survival rates. Treatment decisions in the elderly population are largely driven by performance status and comorbid conditions. The assessment of the impact of comorbidities on overall mortality is possible by using Charlson Comorbidity Index (CCI), which contains a list of 19 diseases whose diagnosis increases the risk of death by at least 20%^9^. Using the age-comorbidity index, we can determine the chance of 10-year survival of patients.

The aim of the study was to assess treatment results of radiotherapy in elderly patients with T1 glottic cancer and to evaluate prognostic factors with particular focus on comorbidities.

## Results

In the analyzed group, median follow-up was 75 months (range 4–284 months). During follow-up 80 patients (61%) died, including 73 patients (56%) of non-oncological causes. In the remaining group of 7 patients (5%) laryngeal cancer was the cause of death.

**Figure 1:**
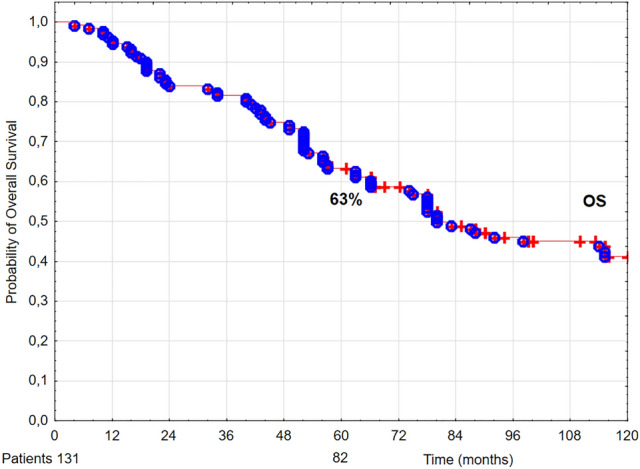
The probability of 5-year overall survival (OS) in T1 glottic cancer patients aged 70 and above.

**Figure 2:**
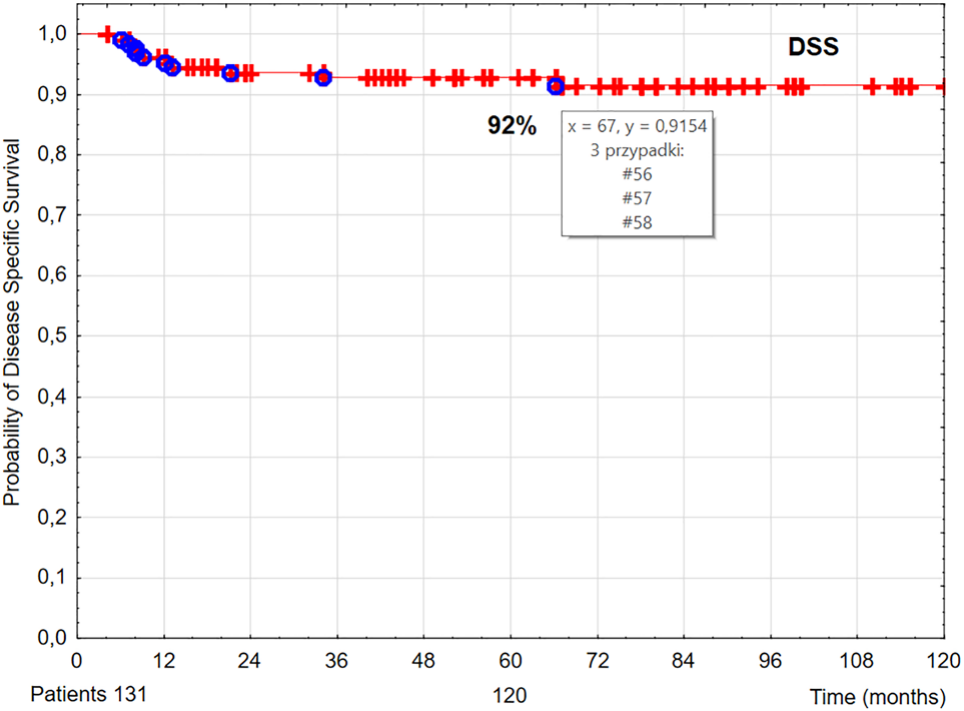
The probability of 5-year disease-specific survival (DSS) in T1 glottic cancer patients aged 70 and above.

**Figure 3:**
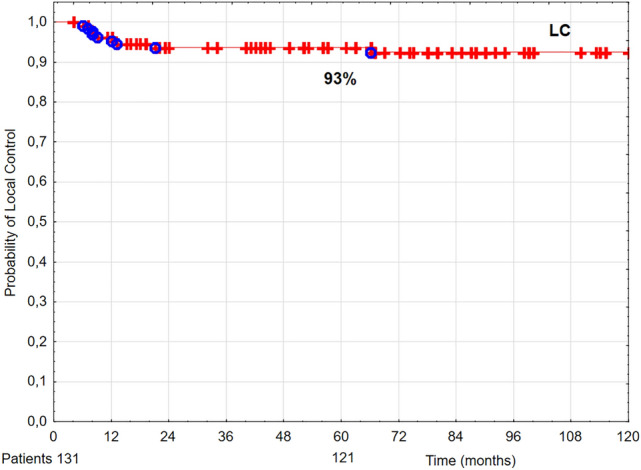
The probability of 5-year local control (LC) in T1 glottic cancer patients aged 70 and above.

Five-year OS, DSS, and LC rates were 63% (Fig. 1), 92% (Fig. 2), and 93% (Fig. 3), respectively.

Nine patients (7%) developed local recurrence; in 5 (4%) patients it was diagnosed within the first year after radiotherapy, whereas in 2 (1.5%) patients within the second year, in 1 (0.75%) patient within the fourth year, and in 1 (0,75%) patient within the fifth year of follow-up. Two patients (1.5%) with local relapse underwent salvage surgical treatment. Seven patients (5.5%) were not subjected to salvage surgery because of advanced disease, poor performance status or patients' refusal.

Overall radiotherapy tolerance was good; 127 patients (97%) completed irradiation according to the treatment plan. Acute and late toxicities were evaluated using the Radiation Therapy Oncology Group (RTOG) scale. Acute mucosal toxicity rates were as follows: G1—75 patients (57%), G2—50 patients (38%), G3—6 patients (5%), while acute skin toxicity rates: G0—10 patients (8%), G1—96 patients (73%), G2—22 patients (17%), G3—3 patients (2%). In 15 patients (11%) late toxicity was observed, including the most frequent: arytenoid edema in 6 patients (5%), vocal cord fibrosis in 3 patients (2%), chronic hoarseness in 6 patients (5%), and xerostomia in 3 patients (2%).

The results of univariate analysis of correlations between selected clinical parameters in relation to 5-year LC, DSS, and OS are presented in Table 1, while the results of univariate analysis of correlations between selected radiotherapy parameters in relation to 5-year LC, DSS, and OS are shown in Table 2.

**Table 1:** The results of univariate analysis of correlations between selected clinical parameters in relation to 5-year local control (LC), disease-specific survival (DSS), and overall survival (OS).

Parameter	Category	5-year LC	5-year DSS	5-year OS
Sex	Male	94%	94%	60%
	Female	80%	80%	80%
		NS	NS	NS
Age	70–74 years	89%	88%	61%
	75–79 years	97%	97%	50%
	80–87 years	100%	100%	63%
		NS	NS	NS
Performance status (ZUBROD)	0–1	89%	89%	71%
	2	95%	94%	58%
		NS	NS	p = 0.046
Tumor stage (T)	T1a	93%	92%	58%
	T1b	83%	83%	70%
		NS	NS	NS
Anterior commissure infiltration	No	94%	94%	63%
	Yes	88%	84%	58%
		NS	NS	NS
Tumor grade (WHO)	G1	93%	93%	62%
	G2	100%	97%	60%
	G3	100%	100%	100%
		NS	NS	NS
Hemoglobin level	≥ 13 g/dl	95%	94%	61%
	< 13 g/dl	77%	77%	40%
		P = 0.022	P = 0.039	NS
BMI (Body Mass Index) (kg/m^2^)	< 18.5	75%	75%	35%
	≥ 18.5 and < 25	96%	95%	60%
	≥ 25	95%	95%	79%
		p = 0.021	p = 0.038	p = 0.016
Cigarettes smoked per day	0	95%	95%	65%
	≤ 20	93%	92%	60%
	> 20	75%	75%	50%
		p = 0.040	p = 0.047	NS
Pack–years	0	96%	96%	65%
	< 40	93%	93%	60%
	≥ 40	91%	90%	55%
		NS	NS	NS
Alcohol abuse	No	95%	94%	61%
	Yes	93%	91%	60%
		NS	NS	NS
Treatment waiting time	≤ 30 days	96%	96%	59%
	> 30 days	82%	76%	70%
		p = 0.020	p = 0.005	NS
Number of comorbidities	1	97%	97%	98%
	2	91%	88%	82%
	3	96%	96%	29%
	4	82%	82%	5%
	5	100%	100%	0%
		NS	NS	p < 0.000
Number of comorbidities	< 3	95%	94%	92%
	≥ 3	92%	92%	19%
		NS	NS	p < 0.000
Charlson Comorbidity Index	5	92%	92%	98%
	6	97%	94%	65%
	7	96%	96%	28%
	8	75%	75%	0%
	9	100%	100%	10%
		NS	NS	p < 0.000
Charlson Comorbidity Index	≤ 6	95%	94%	89%
	> 6	91%	91%	18%
		NS	NS	p < 0.000

*NS* no significance.

**Table 2:** The results of univariate analysis of correlations between selected radiotherapy parameters in relation to 5-year local control (LC), disease-specific survival (DSS), and overall survival (OS).

Parameter	Category	5-year LC	5-year DSS	5-year OS
Radiotherapy technique	I	86%	86%	47%
	II	96%	94%	62%
	III	100%	100%	100%
		NS	NS	p = 0.008
Total dose	≤ 60 Gy	93%	92%	56%
	> 60 Gy	90%	90%	71%
		NS	NS	NS
Fraction size	2 Gy	69%	68%	59%
	2.5 Gy	98%	97%	70%
		p < 0.001	p < 0.001	p = 0.014
Overall treatment time (OTT)	≤ 36 days	98%	97%	65%
	> 36 days	81%	81%	49%
		p < 0.001	p = 0.004	p < 0.001

*NS* no significance.

Univariate analysis performed in the studied group revealed that the following parameters had statistically significant negative effect on LC and DSS rates: hemoglobin level < 13 g/dl, baseline underweight (BMI < 18.5 kg/m^2^), cigarette smoking, in particular > 20 cigarettes per day, treatment waiting time > 30 days (Table 1), fraction dose ≤ 2 Gy, and overall treatment time > 36 days (Table 2).

**Figure 4:**
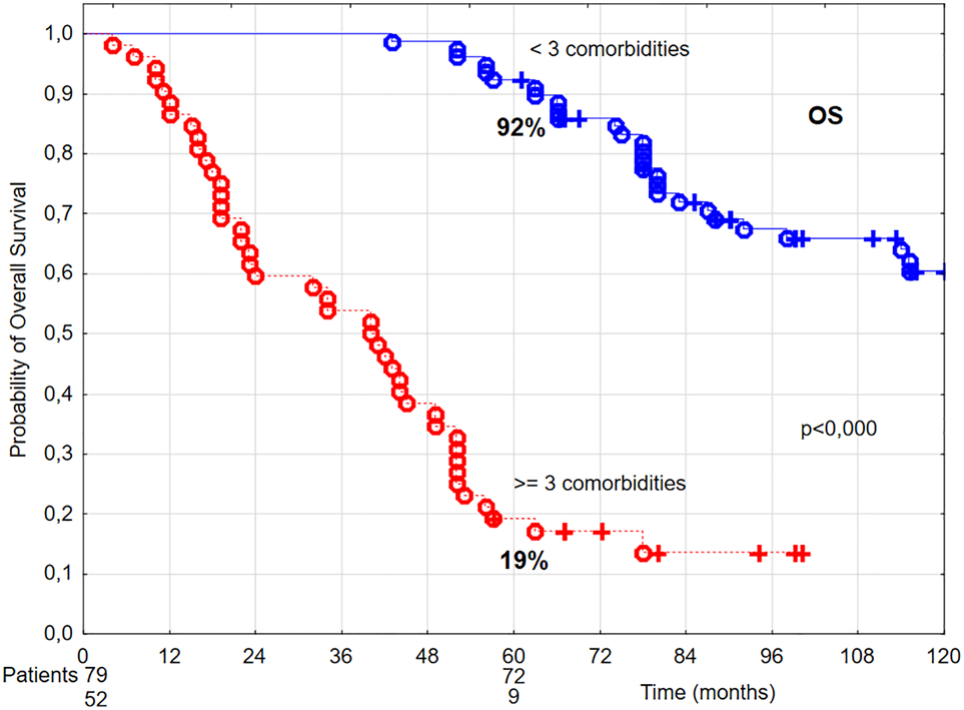
The probability of 5-year overall survival (OS) in T1 glottic cancer patients aged 70 and above in relation to the number of comorbidities.

**Figure 5:**
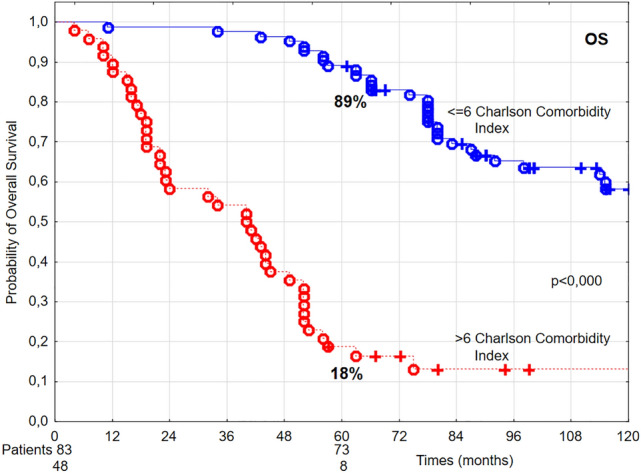
The probability of 5-year overall survival (OS) in T1 glottic cancer patients aged 70 and above in relation to the Charlson Comorbidity Index.

Furthermore, poorer performance status (ZUBROD 2), baseline underweight (BMI < 18.5 kg/m^2^), higher number of comorbidities (Fig. 4), higher CCI (Fig. 5, Table 1), as well as radiotherapy technique, fraction dose ≤ 2 Gy, and overall treatment time > 36 days (Table 2) had statistically significant negative effect on OS.

The results of univariate analysis of correlations between comorbidities in relation to 5-year LC, DSS, and OS are depicted in Table 3. Diabetes in glottic cancer patients from the analyzed group had statistically significant negative impact on LC, DSS, and OS. Patients diagnosed with arterial hypertension, ischemic heart disease, or COPD, or with history of myocardial infarction showed significantly lower 5-year OS.

**Table 3:** The results of univariate analysis of correlations between comorbidities in relation to 5-year local control (LC), disease-specific survival (DSS), and overall survival (OS).

Comorbidity	Category	5-year LC	5-year DSS	5-year OS
Arterial hypertension	No	95%	94%	71%
	Yes	92%	89%	51%
		NS	NS	p = 0.037
Ischemic heart disease	No	94%	94%	80%
	Yes	92%	90%	34%
		NS	NS	p < 0.000
Chronic obstructive pulmonary disease	No	93%	92%	78%
	Yes	94%	93%	39%
		NS	NS	p < 0.000
Peptic ulcer	No	94%	93%	60%
	Yes	92%	92%	56%
		NS	NS	NS
Atherosclerosis	No	94%	94%	65%
	Yes	93%	93%	48%
		NS	NS	NS
Benign prostatic hyperplasia	No	91%	91%	68%
	Yes	97%	97%	50%
		NS	NS	NS
Diabetes	No	96%	95%	68%
	Yes	76%	76%	35%
		p = 0.007	p = 0.012	p = 0.008
History of myocardial infarction	No	93%	92%	68%
	Yes	100%	100%	10%
		NS	NS	p = 0.001
History of stroke	No	93%	92%	60%
	Yes	100%	100%	50%
		NS	NS	NS
Chronic renal failure	No	93%	93%	60%
	Yes	100%	100%	100%
		NS	NS	NS

Multivariate analysis revealed that diabetes, baseline underweight (low BMI), and fraction dose of 2 Gy had statistically significant effect on LC and DSS rates (Tables 4 and 5), whereas many comorbidities, high CCI, baseline underweight, and overall treatment time had negative impact on OS (Table 6).

**Table 4:** The results of multivariate analysis of correlations between selected prognostic factors and local control.

Parameter	Category	N	Relative risk	P value
BMI (Body Mass Index) (kg/m^2^)	≥ 18.5	106	1.00	0.002
	< 18.5	25	1.90	
Fraction size	2.5 Gy	102	1.00	0.002
	2 Gy	29	3.68	
Diabetes	No	111	1.00	0.024
	Yes	20	4.77

**Table 5:** The results of multivariate analysis of correlations between selected prognostic factors and risk of disease-specific survival.

Parameter	Category	N	Relative risk	P value
BMI (Body Mass Index) (kg/m^2^)	≥ 18.5	106	1.00	0.004
	< 18.5	25	1.70	
Fraction size	2.5 Gy	102	1.00	0.001
	2.5 Gy	29	2.95	
Diabetes	No	111	1.00	0.039
	Yes	20	4.05

**Table 6:** The results of multivariate analysis of correlations between selected prognostic factors and overall survival.

Parameter	Category	N	Relative risk	P value
BMI (Body Mass Index) (kg/m^2^)	≥ 18.5	106	1.00	0.029
	< 18.5	25	2.40	
Number of comorbidities	< 3	79	1.00	< 0.001
	≥ 3	52	2.56	
Charlson Comorbidity Index	≤ 6	83	1.00	0.011
	> 6	48	1.64	
Overall treatment time (OTT)	≤ 36 days	80	1.00	< 0.001
	> 36 days	51	1.35	

In the univariate analysis performed, three or more comorbidities were related to a decrease of 5-year OS from 92 to 19% (Fig. 4), while CCI > 6 points was related to the decrease of 5-year OS from 89 to 18% (Fig. 5). Three or more comorbidities were associated with 2.56 times higher death risk, whereas CCI > 6 points was associated with 1.64 times higher death risk.

**Figure 6:**
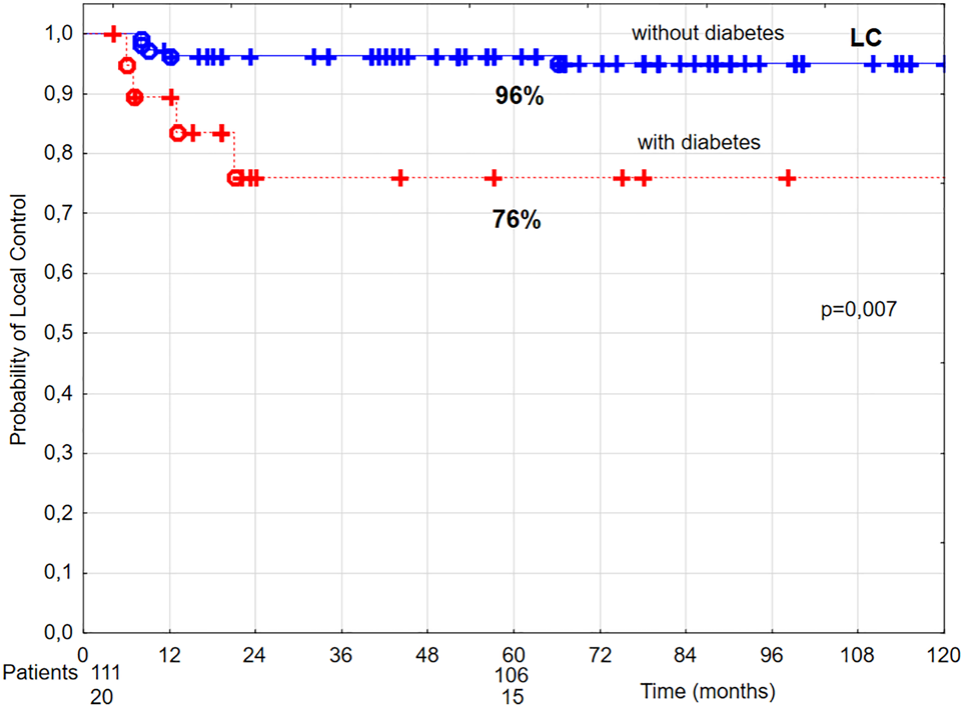
The probability of 5-year local control (LC) in T1 glottic cancer patients aged 70 and above in relation to diabetes.

**Figure 7:**
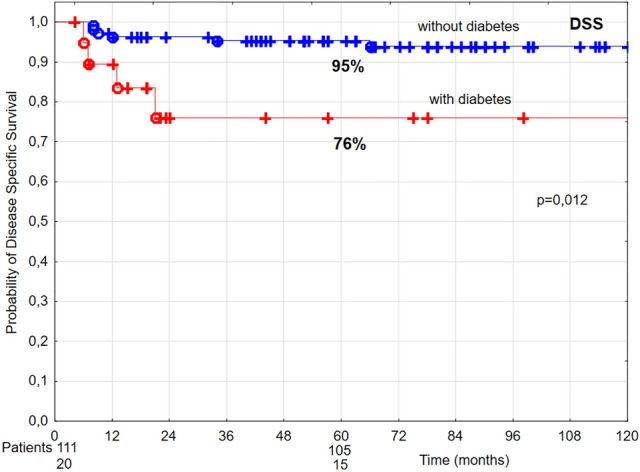
The probability of 5-year disease-specific survival (DSS) in T1 glottic cancer patients aged 70 and above in relation to diabetes.

**Figure 8:**
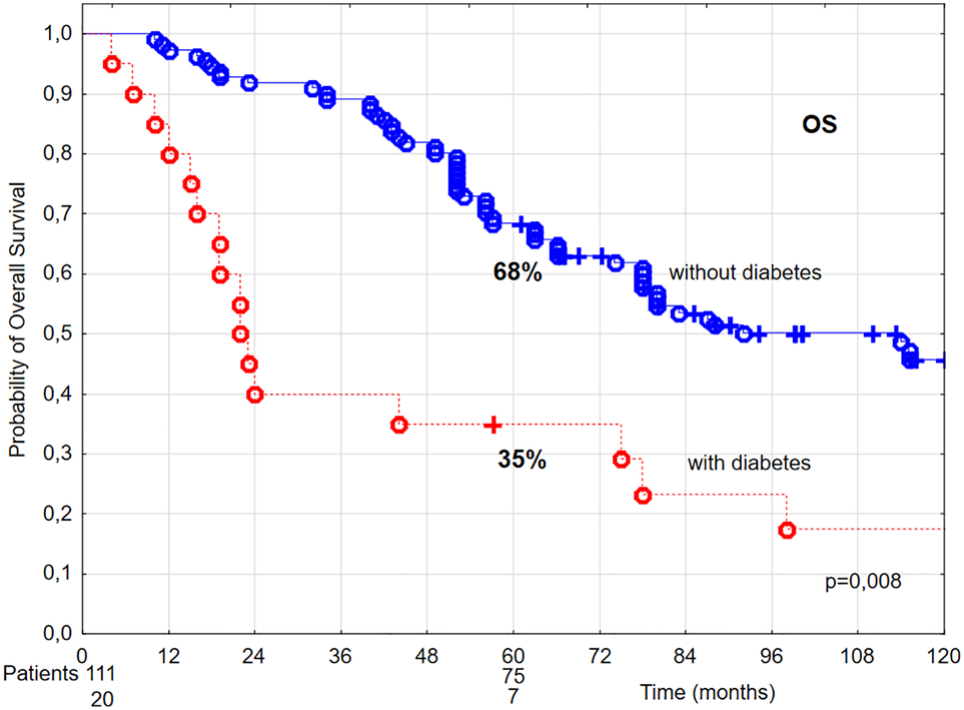
The probability of 5-year overall survival (OS) in T1 glottic cancer patients aged 70 and above in relation to diabetes.

In patients diagnosed with diabetes, in univariate analysis 5-year LC, DSS, and OS were lower by 20%, 19%, and 33%, respectively, compared to patients without diabetes (Figs. 6, 7, 8). Multivariate analysis demonstrated that diabetes was related to 4.77 times higher risk of local recurrence and 4.05 times higher risk of cancer-related death, in comparison to patients without diabetes (Tables 4 and 5).

## Discussion

There are three equivalent treatment methods in early glottic cancer: radiotherapy, laser surgery, and open surgery. Rosier et al. compared the effectiveness of these therapeutic modalities in T1 glottic cancer patients. The study showed 5-year locoregional control and OS rates of 90.6% and 78%, respectively, with no statistically significant differences between the methods applied^10^. Thurnher et al. compared the three treatment approaches in early glottic cancer patients; after 5 years of follow up, DSS was 92% for radiotherapy, 98% for open surgery, and 91% for laser surgery, while recurrence rates were 30%, 13%, and 10%, respectively^11^. In favor of radiotherapy in T1 glottic cancer patients is a very good treatment tolerance, also in older age groups, resulting from the fact that reduced normal tissue volume is exposed to irradiation, because of localized neoplastic process affecting the glottis. In the studied group of patients treated with radiotherapy, both early and late toxicities were low. Other authors also reported that early and late toxicities in elderly head and neck cancer patients treated with radiotherapy were similar to observed in younger age groups^12,13^.

In our group of 131 T1 glottic cancer patients aged 70 and above treated with radiotherapy, after 5 years of follow up the following rates were noted: LC 93%, DSS 92%, and OS 59% (Figs. 1, 2, 3). The obtained LC and DSS rates are similar to those presented in a group of younger patients with T1 glottic cancer treated with irradiation^8,14–16^. On the other hand, the probability of OS in the analyzed group of patients was substantially lower compared to younger age group with the same diagnosis and stage, which is consistent with other authors' results. The researchers showed that age above 65 years had a statistically significantly negative effect on OS in laryngeal cancer patients treated with radiotherapy^8,17,18^. Stokes et al. reported that in a group of early glottic cancer patients age was a negative prognostic factor for death risk; in patients aged between 61 and 70 it was 1.35 times higher than in younger individuals, whereas in patients aged 70 and above—2.29 times higher^19^.

In our group, in univariate analysis numerous factors significantly influenced LC, DSS, and OS (Tables 1, 2, 3).

The results of multivariate analysis confirmed that nutritional status, fraction dose, and diabetes as a comorbidity (Tables 4 and 5) had statistically significant impact on LC and DSS. Furthermore, nutritional status, number of comorbidities, and CCI statistically significantly affected OS (Table 6).

In the present study, baseline underweight (BMI < 18.5 kg/m^2^) was related to 1.9 times higher risk of treatment failure, 1.7 times higher cancer death risk, and 2.4 times higher death risk. In head and neck cancer patients underweight is observed frequently and negatively affects both treatment results and patients' quality of life. The researchers reported that pre-treatment weight loss might be related to higher stage at diagnosis and worse prognosis^20–22^. In a group of 1279 head and neck cancer patients, Gama et al. demonstrated that overweight at diagnosis was related to improved survival in comparison with normal weight. Moreover, underweight patients achieved worse treatment results. BMI at diagnosis was an independent prognostic factor. In their literature review including 8306 head and neck cancer patients, the authors showed the effect of higher BMI on higher OS, lower cancer-related death risk as well as lower recurrence risk compared to normal weight and underweight groups^21^. Zhao-Qu Li et al. noted that BMI had significant influence on laryngeal cancer patients' prognosis; 5-year OS in overweight, normal weight, and underweight groups was 87.2%, 78%, and 34.9%, respectively^22^. Righini et al. showed that in head and neck cancer patients alcohol abuse was a risk factor of underweight^23^. In our group, 44% of patients reported history of alcohol abuse that could favor underweight. For the purpose of the analysis of nutritional status we used BMI, which is a parameter widely adopted by many researchers. Nutritional status plays an important role in the decision-making process on management. Elderly patients frequently suffer from malnutrition, therefore, Comprehensive Geriatric Assessment (CGA) includes nutritional status evaluation using Mini Nutritional Assessment (MNA). The assessment of nutritional status is based on a combination of anthropometric measurements, BMI evaluation, and collecting information on current dietary habits including the quantity and quality of consumed food. Using MNA in practice allows for detecting nutritional disorders before weight loss or protein deficiency occurs and for early implementation of nutritional interventions^24^.

Another identified independent prognostic factor was fraction dose (Tables 4 and 5). In patients who received fraction dose of 2 Gy, the risk of local failure was 3.68 times higher, while cancer death risk was 2.95 times higher than in patients receiving fraction dose of 2.5 Gy. Other researchers also reported that higher fraction dose improved radiotherapy results in early glottic cancer patients^8,25–28^.

Mendenhall et al., analyzing a cohort of T1 glottic cancer patients treated with hypofractionated radiotherapy, noted 5-year LC in 94% of cases in comparison with 80% treated with conventional radiotherapy^25^. A prospective study of 180 patients with T1 glottic cancer by Yamazaki et al. revealed a 15% gain in 5-year LC in a group of patients who received fraction dose of 2.25 Gy compared to a group receiving a dose of 2 Gy; both doses showed similar toxicity^26^. Moreover, a retrospective analysis of 157 T1 glottic cancer patients by Kim et al. reported an improvement of 11% in 5-year DSS in a group receiving fraction dose of 2.25 Gy compared to a group receiving a dose of 2 Gy^27^. Bledsoe et al. showed a statistically significant gain of 2.2% in 5-year OS in a large group of 10,212 T1 glottic cancer patients treated with hypofractionated radiotherapy^28^. In irradiated patients with T1 glottic cancer, Ermis et al. carried out radiobiological considerations aiming to explain treatment outcome improvement in a group receiving hypofractionated radiotherapy. The authors calculated the biologically effective dose (BED) incorporating treatment time for different fractionation methods in glottic cancer patients (assumed α/β ratio ≥ 10 Gy) and received the following values: for total dose of 55 Gy in 20 fractions BED = 67 Gy, for total dose of 70 Gy in 35 fractions BED = 64.7 Gy, for total dose of 60 Gy in 24 fractions BED = 67.3 Gy, while for total dose of 50 Gy in 15 fractions BED = 68.9 Gy. They proved that tumor BED values were similar. However, BED calculated for tissues reacting with late toxicities (assumed α/β = 3) for total dose of 55 Gy in 20 fractions was 105.4 Gy, while for total dose of 70 Gy in 35 fractions—116.6 Gy. The observed difference might serve as explanation of the therapeutic gain of hypofractionation^29^. In patients with T1 glottic cancer, higher fraction doses were well tolerated by older individuals because of limited irradiated volume^8,29^. Using hypofractionation in patients with early glottic cancer reduces overall treatment time (OTT) by 2–3.5 weeks, therefore, repopulation that starts around 4th week of radiotherapy has lower impact on treatment outcome. The researchers postulate that in hypofractionated radiotherapy shorter OTT rather than fraction dose results in superior treatment effects^30^. Yamazaki et al. pointed out the financial aspect of reduced radiotherapy duration in patients with T1 glottic cancer^31^. For elderly patients both shorter OTT and shorter hospitalization time are relevant.

Head and neck cancer patients frequently suffer from comorbidities related to long-term tobacco and alcohol abuse which include COPD, cirrhosis, and cardiovascular disorders^32,33^.

In our group of glottic cancer patients aged 70 and above all patients had 1–5 comorbidities. In a cohort of 310 head and neck cancer patients above 70 years of age, Sanabria et al. noted comorbidities in 75% of cases^32^. Paleri et al. conducted a retrospective survey on a group of 180 patients with laryngeal cancer, founding comorbidities, most frequently cardiovascular and respiratory, in 65% of cases^33^. Both groups of authors used Adult Comorbidity Evaluation—27 (ACE-27) to assess comorbid diseases; it is a complex instrument that encodes all possible types of comorbidities in head and neck cancer patients, excluding malnutrition^34^. For the purpose of our analysis, CCI was used to evaluate the impact of comorbidities^9^. Charlson Comorbidity Index includes 19 conditions that increase the death risk by at least 20%. Each condition is assigned a weight from 1 to 6 points, depending on the influence on survival. The age-comorbidity score shows the probability of surviving 10 years. In the current study, it was demonstrated that 5-year OS decreased with increasing number of comorbidities from 98% in the presence of 1 comorbidity to 82% in the presence of 2 comorbidities, 29% in the presence of 3 comorbidities, 5% in the presence of 4 comorbidities, and, finally, 0% in the presence of 5 comorbidities. The results of multivariate analysis confirmed an independent effect of the number of comorbid diseases on OS; patients with at least 3 comorbidities had 2.55 times higher death risk than patients with lower number of comorbidities. Comorbid conditions, regardless of their number, influenced neither LC nor DSS rates. In a group with CCI of 5–6 points the probability of 5-year survival was 89%, while in a group with 7–9 points it reached only 18%. Multivariate analysis showed that CCI > 6 was related to 1.64 times higher death risk.

In our material it was shown that higher number of comorbidities was associated with higher probability of the patient dying from causes other than cancer, what corresponds with other authors’ findings^35–39^.

Sabin et al. analyzed the effect of comorbidities in laryngeal cancer patients using CCI^37^. They noted a median survival of 41 months in a group of low CCI and 8 months in a group of high CCI. The authors reported that CCI was a strong prognostic factor for survival in patients with laryngeal cancer and concluded that elderly patients with many comorbidities would die from causes other than laryngeal cancer.

Some authors also showed higher risk of relapse and cancer death in patients with many comorbidities, however, in our material this relation was not observed^36,40^. It could be explained with the fact that patients with many comorbid conditions receive suboptimal treatment due to concerns about toxicity and, for this reason, experience inferior treatment outcomes. Another explanation could be the fact that intense treatment is not well tolerated and promotes treatment discontinuities that diminish its effectiveness. A meta-analysis revealed lower benefit from intense treatment of elderly patients^41^. Anticancer treatment frequently exacerbates the signs and symptoms of comorbidities, which can negatively impact treatment outcomes and increase the risk of severe complications^42^. The intensification of treatment should be confined to thoroughly selected patients and carried out when necessary. In our patients treatment tolerance was good because of low irradiated volume.

In our material, type 2 diabetes as comorbidity was related to significantly lower 5-year LC, DSS, and OS rates (Table 3). Diabetic patients above 70 years of age had 4.77 times higher treatment failure risk and 4.05 times higher cancer-specific death risk (Tables 4 and 5). Numerous studies show relation between diabetes (particularly type 2 diabetes) and higher incidence of many cancers, including pancreatic, colorectal, liver, urinary bladder, endometrial, breast, and kidney cancers as well as non-Hodgkin lymphomas^43–50^. It was also demonstrated that patients with diabetes have higher risk of cancer death^44,51^.

There are certain etiological factors, including overweight, obesity, diet, age, physical activity, alcohol and tobacco use, that are common for both diabetes and cancer. Higher cancer risk in diabetic patients can be related to the following metabolic disorders: hyperglycemia, insulin resistance, hyperinsulinemia, and elevated level of insulin-like growth factor type 1 (IGF-1). It was observed that in tumor cells of certain cancers expression of insulin receptor, which promotes proliferation, is higher. Both insulin and IGF-1 have mitogenic effect and stimulate cell proliferation. Additionally, in the process of carcinogenesis chronic inflammation can be relevant^52–54^. Patients treated with insulin have higher risk of developing cancer, however, no evidence is available to confirm carcinogenic effect of the hormone^55^. Neoplastic cells show higher metabolic activity and use glucose as their basic source of energy, therefore, it is possible that cancer growth depends on glucose availability in the body. Diabetes promotes progression of atherosclerosis and increases the risk of cardiovascular incidence, what can have a negative effect on patients’ survival. Furthermore, occurring microcirculation disorders can impair tumor angiogenesis and, consequently, decrease tumor radiosensitivity and probability of curative effect of radiotherapy.

## Methods

A retrospective analysis was performed in a group of 131 patients aged 70 and above with T1N0M0 glottic cancer, representing 23% of all patients with this type and stage of cancer treated with radiation therapy at the National Institute of Oncology in Cracow between 1977 and 2007. All patients gave written informed consent to the proposed treatment and the use of medical data for research. The study was performed in accordance with the guidelines and regulations of the Polish National Cancer Institute, which are consistent with the Declaration of Helsinki and good clinical practice. This retrospective analysis was also approved by the Ethical Committee at the Regional Medical Chamber in Cracow (Poland). The analyzed group included 121 men (92%) and 10 women (8%). The mean age was 74 years (range, 70–87 years). For the purpose of the study the patients were divided into the following three subgroups according to age: I subgroup—70–74 years of age—83 patients (63%), II subgroup—75–79 years of age—37 patients (28%), and III subgroup—80–87 years of age—11 patients (9%). Performance status of the majority of patients was very good or good (ZUBROD 0–1) —98 patients (75%), whereas in 33 patients (25%) it was assessed as ZUBROD 2.

One hundred and eighteen patients (90%) were diagnosed with T1a glottic cancer, while in 13 patients (10%) cancer was staged as T1b. In 34 cases (26%) the tumor involved anterior commissure. All patients were diagnosed with squamous cell carcinoma, including 66 cases (50%) of grade 1, 47 (36%) cases of grade 2, and 1 case (1%) of grade 3 cancer. In 17 patients (13%) grade was not assessed.

The mean treatment waiting time, defined as the time from obtaining tumor sample to beginning of radiotherapy, was 51 days (range, 8–145 days).

The mean baseline hemoglobin level was 14.6 g/dl (range, 9.8–16.7 g/dl). In 22 patients (17%) it did not exceed 13 g/dl, whereas in 109 patients (83%) it was 13 g/dl or higher.

Nutritional status was assessed based on patients' histories using body mass index (BMI) with the following thresholds adopted: < 18.5 kg/m^2^—underweight, ≥ 18.5 kg/m^2^ and < 25 kg/m^2^—normal weight, ≥ 25 kg/m^2^—overweight. There were 83 patients (63%) with normal weight, while 25 patients (19%) were underweight and 23 patients (18%)—overweight.

In the analyzed group only 35 patients (27%) did not have a history of tobacco smoking; the remaining smoked 2–50 cigarettes per day (mean, 14 cigarettes per day). The mean time of tobacco use was 39 years (range, 10–67 years) and mean lifetime tobacco exposure was 38 pack-years (range, 8–144). Alcohol abuse was reported by 58 patients (44%), which for men meant consuming 15 or more drinks per week, while for women—8 or more drinks. Clinical characteristics of patients is presented in Table 7.

**Table 7:** Clinical characteristics of patients with T1 glottic cancer aged 70 and above.

Parameter	Category	Number of patients, n (%)
Sex	Male	121 (92%)
	Female	10 (8%)
Age	70–74 years	83 (63%)
	75–79 years	37 (28%)
	80–87 years	11 (9%)
Performance status (ZUBROD)	0	5 (4%)
	1	93 (71%)
	2	33 (25%)
Tumor stage (T)	T1a	118 (90%)
	T1b	13 (10%)
Anterior commissure infiltration	No	97 (74%)
	Yes	34 (26%)
Tumor grade (WHO)	G1	66 (50%)
	G2	47 (36%)
	G3	1 (1%)
	GX	17 (13%)
Hemoglobin level	≥ 13 g/dl	109 (83%)
	< 13 g/dl	22 (17%)
BMI (Body Mass Index) (kg/m^2^)	< 18.5	25 (19%)
	≥ 18.5 and < 25	83 (63%)
	≥ 25	23 (18%)
Cigarettes smoked per day	0	35 (27%)
	≤ 20	61 (46%)
	> 20	35 (27%)
Pack-years	0	35 (27%)
	< 40	15 (11%)
	≥ 40	81 (62%)
Alcohol abuse	No	73 (56%)
	Yes	58 (44%)
Treatment waiting time	≤ 30 days	30 (23%)
	> 30 days	101 (77%)

*GX* grade was not assessed.

In the studied group comorbid diseases were present in all patients; 32 patients (24%) had one comorbidity, 47 patients (36%)—two comorbidities, 28 patients (22%)—three, 17 patients (13%)—four, while 7 patients (5%)—five comorbidities. The most common comorbid conditions were: arterial hypertension, ischemic heart disease, and chronic obstructive pulmonary disease (COPD) (Table 8).

**Table 8:** Comorbidities in patients with T1N0M0 glottic cancer.

Disease	Number of patients	Percentage (%)
Arterial hypertension	63	48
Ischemic heart disease	57	44
Chronic obstructive pulmonary disease	57	44
Peptic ulcer	37	28
Atherosclerosis	32	24
Benign prostatic hyperplasia	31	23
Diabetes	20	15
History of myocardial infarction	9	7
History of stroke	8	6
Chronic renal failure	7	5

As part of the study, an analysis of the impact of comorbidities on overall survival in each patient was performed using Charlson Comorbidity Index (CCI)^9^. The results obtained were as follows: 5 pts—48 patients (37%), 6 pts—35 patients (27%), 7 pts—26 patients (20%), 8 pts—13 patients (10%), 9 pts—9 patients (7%).

Three radiotherapy techniques were applied in the analyzed group: (I) two oblique wedged beams of cobalt 60 or 6 MV linac photons in 82 patients (62%), (II) two opposite wedged beams of cobalt 60 or 6 MV linac photons in 47 patients (36%), and (III) single mixed photon–electron beam in 2 patients (2%). Single beam was used in patients with degenerative spine disorders who could not hold therapeutic position with head bent backwards.

All patients were irradiated with 1 fraction dose daily, 5 times a week, to a total dose of 60–70 Gy (mean, 61 Gy) with fraction dose of 2 or 2.5 Gy. Mean overall treatment time was 38 days (range, 31–57 days). Table 9 summarizes the distribution of clinical data with regard to irradiation parameters.

**Table 9:** Clinical data distribution with regard to irradiation parameters in T1 glottic cancer patients aged 70 and above.

Parameter	Category	Number of patients, n (%)
Radiotherapy technique	(I) oblique wedged beams	82 (62%)
	(II) opposite wedged beams	47 (36%)
	(III) mixed photon–electron beam	2 (2%)
Total dose	≤ 60 Gy	110 (84%)
	> 60 Gy	21 (16%)
Fraction size	Normofractionated (2 Gy)	29 (22%)
	Hypofractionated (2.5 Gy)	102 (78%)
Overall treatment time (OTT)	≤ 36 days	80 (61%)
	> 36 days	51 (39%)

The probabilities of 5-year overall survival (OS), disease-specific survival (DSS), and local control (LC) were calculated using Kaplan–Meier method. The log-rank and chi-square tests were applied do assess statistical differences between groups; the level of statistical significance p < 0.05 was adopted. Independent prognostic factors were identified using multivariate Cox analysis.

## Conclusions

Radiotherapy is an effective treatment modality in elderly T1 glottic cancer patients. Diabetes, underweight, and conventional dose fractionation decrease the probability of curative effect of radiotherapy in elderly glottic cancer patients. High number of comorbid diseases diminishes the probability of long-term survival in elderly glottic cancer patients.
